# Training the next generation of global health experts: experiences and recommendations from Pacific Rim universities

**DOI:** 10.1186/s12992-016-0162-z

**Published:** 2016-06-23

**Authors:** Mellissa Withers, David Press, Heather Wipfli, Judith McCool, Chang-Chuan Chan, Masamine Jimba, Christopher Tremewan, Jonathan Samet

**Affiliations:** Department of Preventive Medicine, University of Southern California, Keck School of Medicine, 2001 N Soto Street SSB 318G, Los Angeles, CA 90064 USA; School of Population Health, University of Auckland, Auckland, New Zealand; National Taiwan University (NTU) College of Public Health, Global Health Center, Taipei City, Chinese Taipei; Department of Community and Global Health, University of Tokyo, Graduate School of Medicine, Tokyo, Japan; Association of Pacific Rim Universities, Hong Kong, Hong Kong SAR

**Keywords:** Global health education, Training, Pacific Rim, Global health degrees, MPH, Academic programs

## Abstract

**Background:**

Finding solutions to global health problems will require a highly-trained, inter-disciplinary workforce. Global health education and research can potentially have long-range impact in addressing the global burden of disease and protecting and improving the health of the global population.

**Methods:**

We conducted an online survey of twelve higher education institutions in the Pacific Rim that spanned the period 2005–2011. Program administrators provided data on program concentrations, student enrollment and student funding opportunities for 41 public health programs, including those specific to global health.

**Results:**

The Master of Public Health (MPH) was the most common degree offered. A growing demand for global health education was evident. Enrollment in global health programs increased over three-fold between 2005–2011. Very few institutions had specific global health programs or offered training to undergraduates. Funding for student scholarships was also lacking.

**Conclusions:**

The growing demand for global health education suggests that universities in the Pacific Rim should increase educational and training opportunities in this field. Schools of medicine may not be fully equipped to teach global health-related courses and to mentor students who are interested in global health. Increasing the number of dedicated global health research and training institutions in the Pacific Rim can contribute to building capacity in the region. Faculty from different departments and disciplines should be engaged to provide multi-disciplinary global health educational opportunities for undergraduate and graduate students. New, innovative ways to collaborate in education, such as distance education, can also help universities offer a wider range of global health-related courses. Additional funding of global health is also required.

## Background

Increasingly, we are recognizing and responding to our global interdependence and collective exposure to transnational threats to health, as well as appreciating the relevance of global forces such as trade policy, migration, cooperation and governance [1, 2]. The solutions to current and future global health problems will require a highly-trained, inter-disciplinary global health workforce from the public and private sectors, non-governmental organizations and governments. Successful efforts to address emerging global health challenges will require the coordination of global health education, surveillance, research and service. However, many scholars have highlighted the lack of a sufficient workforce of trained global health professionals that will be able to accomplish these tasks [1–8].

Koplan et al. define global health as “the term applied to a rapidly growing area of research, training, and application of public health on a global scale” and point out that global health expertise requires not only public health training, but also additional competencies in topics such as global governance, cultural sensitivity, global disease trends, and global development, among others [9]. Although global health inherently has unbounded scope, its priorities are governed not only by global influences but by regional factors. The economies of the Pacific Rim, the lands around the edges of the Pacific Ocean, are pivotal in terms of their contributions to the global burden of disease [10]. More than 40 economies belong to this diverse region, representing Asia, Australia and Oceania, and the Americas. Key health issues facing the region include climate change, natural disasters, environmental degradation, the rise of non-communicable diseases, and the threat of emerging and re-emerging diseases, such as SARS and tuberculosis. The health of Pacific Rim populations is challenged by some common issues, including fast-paced economic growth and industrialization, demographic changes, particularly aging, and rapid and extensive urbanization. The various health systems, while in differing phases of development, in some cases are struggling to provide equitable and fair health care for all, especially given the market-driven approaches and wide economic and health disparities.

Global health education and research can potentially have long-range impact in addressing the global burden of disease and protecting and improving the health of the global population. Many universities, institutes and associations around the world now offer global health training programs and numerous descriptions of such programs in the United States have been offered in the literature by such institutions as Boston University, University of California at San Francisco and Emory University [11–23]. Generally, such programs aim to provide students with opportunities to gain hands-on experience working in developing economies, improve core competencies, and gain increased awareness of the health challenges faced by populations around the world [24, 25]. Most of these programs are housed within a larger health or public health program that is not focused specifically on global health. Furthermore, many of these global health experiences are short in duration, and offer limited experiences and exposure to the complex and often highly political and economic drivers of global health inequities. For example, Lencucha and Mohindra [26] examined global health courses at universities in Canada and the U.S. through a web search and found 67 courses were offered at 31 universities in the 2008–2009 academic year. They called for a more interdisciplinary training outside of traditional health science disciplines and highlighted the difficulty of including the breadth of issues pertaining to global health in a single course [26]. Several other scholars including Ackerly et al. [2], Merson et al. [23] and Drain et al. [24] have also called for a more comprehensive and ethical approach to global health training.

Here we present an analysis of the educational and training programs related to public health, focusing on global health, offered by numerous academic institutions in the Pacific Rim. We highlight recent trends in the increasing demand for global health education. We discuss the role played by academic institutions in the Pacific Rim in providing educational opportunities in global health and make recommendations on how universities can be better equipped to meet the demand for future global health leaders.

### Background on APRU

The Association of Pacific Rim Universities (APRU) is an international consortium of 45 prestigious research universities in the Pacific Rim, representing 16 economies, 130,000 faculty members and more than two million students. The APRU Global Health Program was launched in 2007–08. The Program’s activities in research, training, and service around the global illustrate the diverse dimensions of global health. The members are faculty who are actively engaged in global health research and education. A main purpose of the program is to foster further discussion on global health in a regional context, as our institutions respond to global and regional needs for capacity building. One key element is global health education and training, which takes place at the undergraduate, graduate and post-graduate levels across the institutions. The main objective of this study is to investigate the practices in educating and training the next generation of global health professionals in the Pacific Rim.

## Methods

We conducted an online survey of APRU member and partner institutions in 2012. An invitation was sent out through the APRU listserv and by private email invitation. One administrator from each university’s admissions office was asked to complete the written survey regarding the post-graduate public health programs that were offered and which were specific to global health. The survey was composed of three sections. Section 1 related to global health programs. Six questions were included, such as “What programs specific to global health do you offer?” and “Do you offer any online global health-related courses? Section 2 included fifty questions related to student enrollment in each of the programs. Questions included “How many students are enrolled in this program?” and “How many foreign students are enrolled in this program?” Section 3 contained eight questions focused on funding, such as “What percentage of students receive grants?” and “Are university scholarships available to your students?”

A public health program was defined as any general degree program degree or certificate program relating to public health, while a *global health* program was defined broadly as any program associated with an international or a global focus (including a degree program or a global health track within another degree program), regardless of the actual name of the program. In order to track trends, the survey asked about two time points: 2005 and 2011.

## Results

Twelve universities from Australia, Chinese Taipei, Hong Kong, Indonesia, Japan, New Zealand, Singapore, the United States, and Vietnam^1^ provided programmatic and student data on 41 postgraduate programs in public health, including five institutions offering postgraduate programs specific to global health.

### The programs

A rapidly growing demand for post-graduate public health education was evident from our findings. Over two-thirds of the academic programs surveyed were created after 1990, with the greatest number of new programs in the previous five years. As seen in Table 1, the Masters of Public Health (MPH) Program was the most common academic program offered across institutions (100 %), followed by the Doctor of Philosophy (PhD) or another Doctoral Program (91.7 %), and the Masters of Science (MS) Program (58.2 %). Aside from the most common degrees of MPH, PhD, and DrPH, some universities offered degrees specific to global health, such as a Master of International Public Health, and several schools offered graduate diplomas or graduate certificate programs in global health. Programs were mainly housed within schools of public health but some universities housed their programs within the school of medicine or, in one case, the school of public policy. Of the 12 universities surveyed, only four offered distance-learning courses in public health and only five offered the opportunity for undergraduates to take any courses in public health.

**Table 1:** Summary of APRU academic programs

University Name	MPH program	MS program	PhD or doctoral program	Certificate training program	Other	Postgrad global health specific program	Online courses	Under grad global health classes
Chinese University of Hong Kong	Yes	Yes	Yes	No	No	No	No	Yes
Hanoi School of Public Health	Yes	No	Yes	Yes	Yes	No	No	No
John Hopkins University	Yes	Yes	Yes	Yes	No	Yes	Yes	Yes
National Taiwan University	Yes	Yes	Yes	Yes	No	No	No	Yes
Seoul National University	Yes	No	Yes	No	No	No	Yes	No
Singapore National University	Yes	No	Yes	No	Yes	No	No	No
University of Auckland	Yes	No	No	No	Yes	Yes	No	No
University of Indonesia	Yes	No	Yes	Yes	No	No	Yes	Yes
University of Malaya	Yes	Yes	Yes	Yes	Yes	No	No	No
University of Southern California	Yes	Yes	Yes	No	No	Yes	No	Yes
University of Sydney	Yes	Yes	Yes	Yes	Yes	Yes	Yes	No
University of Tokyo	Yes	Yes	Yes	No	No	Yes	No	Yes
Total	12	7	11	6	5	5	4	6

### The students

As seen in Table 2, the overall student enrollment in post-graduate public health programs increased approximately 21 % from 2005 to 2011. The highest percentage change increases were observed within the global health programs, which had an increased enrollment of 351 %. The slowest growth during that same time period was found within doctoral programs (6 %). In terms of student demographics, the majority of students being trained in each academic program in 2005 were female, and this imbalance became even more pronounced between 2005–2011, when more females enrolled in Doctorate, MS and Global Health programs. For example, in 2011, 77 % of postgraduate global health students were female. MPH students accounted for the biggest proportion of student body among all degrees.

**Table 2:** Frequency distribution and percentage change of student characteristics aggregated across APRU institutions from 2005 to 2011, by academic program

	Total^a^			MPH			Doctorate			MS			Global health		
	2005	2011	(%chg)	2005	2011	(%chg)	2005	2011	(%chg)	2005	2011	(%chg)	2005	2011	(%chg)
Student enrollment	3314	4009	21.0 %	1504	1874	24.6 %	998	1055	5.7 %	704	944	34.1 %	109	491	350.5 %
Sex^b^															
Female	2202	2760	25.3 %	1030	1266	22.9 %	631	715	13.3 %	459	646	40.7 %	83	377	354.2 %
Male	1112	1249	12.3 %	474	608	28.3 %	367	340	−7.4 %	245	298	21.6 %	26	114	338.5 %
Student status															
International	727	1014	39.5 %	420	596	41.9 %	207	231	11.6 %	61	127	108.2 %	39	150	284.6 %
Domestic	2587	2995	15.8 %	1084	1278	17.9 %	791	824	4.2 %	643	817	27.1 %	70	341	387.1 %
Enrollment status															
Full-time	2473	2974	20.3 %	938	1058	12.8 %	832	931	11.9 %	638	887	39.0 %	66	361	447.0 %
Part-time	841	1035	23.1 %	566	816	44.2 %	166	124	−25.3 %	66	57	−13.6 %	43	130	202.3 %
Physician (MD) status															
Non-MDs	‡			1114	1381	24.0 %	‡			‡			‡		
MDs				390	493	26.4 %									

^a^ Column totals do not sum to 100 % due to overlapping Global Health programs; Total student enrollment reflects sum of collected fields

Total for sub-categories reflects imputation based on the overall proportion of collected fields

^b^ Derived based on proportion of females in each program. Missing data fields were imputed based on the overall proportion from the programs with complete data; <1 % of students were imputed

‡ Missing data for two or more Universities

An increasing flow of international students across universities was also observed. Between 2005 and 2011, the number of international students enrolled increased 40 % (from *n* = 727 to *n* = 1014) in general public health programs and 285 % (from *n* = 39 to *n* = 150) in global health programs specifically. For both time periods, at least one in five students enrolled in MPH, Doctorate and Global Health Programs were international students. Proportional increases in international student matriculation outpaced those of domestic students from 2005–2011 in all programs except those specific to global health, in which an increase of 387.1 % was found in domestic student matriculation.

### Funding

Only one-half of universities offered scholarships for students. Almost every school said that national grants were available to students. However, one-half of universities reported that only a small minority of their students (1-25 %) received such grants. All of the universities reported little to no growth with regard to funding for students between 2005 and 2011.

## Discussion

Global health education is becoming increasingly visible and the proliferation of new programs in the region shows that interest and participation in global health education specifically has accelerated in recent years. Merson and Chapman Page attribute this growth in the United States to three factors, including greater emphasis on internationalization in higher education, heightened public visibility of the global health agenda, and the expansion of new resources and opportunities for universities and students [27]. This trend has also been emphasized in the literature by Merson [23], Drain et al. [24] and Crump and Sugarman [25].

Our findings suggest that the growing demand for global health education and training programs is likely to continue. A preference for practice-based degrees, such as the MPH, over the traditional research PhD degree was evident in our study. However, very few schools had established dedicated departments, or centers, of global health and very few offered undergraduate courses in global health. However, our results show that global health programs can be included in a wide variety of disciplines and housed in many different types of schools (e.g., medical, public health, nursing), which reflects the trend towards a more multidisciplinary global health field [6].

Here, we offer four major recommendations on providing global health education and training based on our collective experiences in training and educating students in this field, considering the special challenges that universities without schools of public health may face. First, the proliferation and the diversity of global health education programs in the Pacific Rim suggest that universities should meet the growing demand for global health education by offering more programs and degrees focused specifically on global health. Increasing the number of dedicated global health research institutions in the Pacific Rim could contribute to building capacity in the region. We found that very few schools in our study had established dedicated departments, or centers of global health. Many public health-related degrees were offered in schools of medicine, which may not be equipped to teach global health-related courses. One important challenge is the lack of teaching capacity in public health in general and global health in particular, especially in the smaller programs. Faculty may not be prepared to teach the diversity of topics required in global health education. Furthermore, schools of medicine may not have enough faculty members equipped to mentor students who are interested in global health.

Combined degree programs with a global health track, concentration or specialization, such as MD/PhD and MD/MPH programs, could help meet the demand for global health training in multidisciplinary programs. Institutions that do not have schools of public health should consider incorporating global health courses into already existing degree programs, such as medical degrees. In addition, by offering certificate programs in global health, students could pursue training in global health without participating in a full public health degree program. Universities that already offer general public health degrees should add core courses in global health to afford the opportunity for their students to broaden their training into global health.

Second, we also found that very few programs in our study offered undergraduate courses in global health. We encourage universities to offer more global health courses that undergraduate students can join. Exposing undergraduates to global health courses may help spark students’ interest in global health early in their academic careers and encourage them to pursue graduate training in this field. At the University of Southern California, there has been substantial demand from undergraduates for global health courses and opportunities over the last five years. Similarly, in other institutions, the introduction of global health as part of the undergraduate medical training is beginning to emerge, although it is likely to face challenges in terms of perceived relevance by pre-clinical and clinical students and program overload. We suggest that global health problems represent a superb platform for teaching interdisciplinarity.

While globalization has brought challenges for health, it has also brought new ways to collaborate in education. New, more flexible models of learning should be embraced, such as distance learning and experiential learning opportunities [5]. These can provide another way that universities can offer a wider range of global health courses without additional expenses. Curricular materials can be shared; lectures can be webcast globally; and multi-institutional research is increasingly feasible. Universities can take advantage of online courses offered for free, such as those offered by The Johns Hopkins Bloomberg School of Public Health or the Consortium of Universities for Global Health (CUGH). Faculty from two APRU member universities in the United States (the University of California at Irvine and the University of Southern California) developed an online course in global health leadership in which five universities from around the Pacific Rim participated. Students can also be encouraged to enroll in massive open online courses (MOOC). However, careful attention must be paid to ensure that contact hours within these courses remain as high as in traditional full-time programs and that quality instruction is maintained [6, 24, 28–31].

Third, there is a growing recognition that improving the health of populations across the globe will require a multi-faceted, multi-disciplinary approach, as highlighted in the literature [1, 4, 5, 21, 22, 24]. The factors which determine health status are complex and global health training requires an understanding of the multitude of determinants of health, such as social, economic, political and demographic factors. Preparing a global health workforce with expertise in an array of disciplines and contexts can help address global health challenges. Training of more than just medical doctors or epidemiologists will be required to meet these challenges. As the world becomes increasingly globalized through international travel and commerce, it is necessary to think about health in a global context. Improving global health can support national, regional and global security interests by fostering political stability, diplomacy, and economic growth worldwide. Experts in trade, migration, security and governance can help promote health globally. Solutions to combat the rise in non-communicable diseases and the influence of the environment in health status require the application of theories and knowledge from multiple fields. The research capabilities and surveillance required to combat pressing global health problems require significant capacity at many levels, including governmental, research and clinical. In addition, the ability of universities to conduct research requires the existence of capable Institutional Review Boards (also called Research Ethics Committees) that possess an advanced level of public health ethics, research methods, as well as knowledge from many other disciplines, in order to protect human subjects involved in research within the country, as well as outside. Diversity within schools in terms of programs and disciplines can promote training at many levels.

Universities are encouraged to involve faculty from different departments and disciplines in providing global health education. Courses from a variety of disciplines should be offered, such as courses in medical anthropology, health economics, environmental health, psychology, demography, big data and health informatics, business and leadership, and global health diplomacy. In addition, the role of cultural beliefs and practices in health outcomes is well-acknowledged and calls for training in qualitative methods. A major gap in many students’ training is the absence of qualitative research methods courses, especially among medical students.

Fourth, our results corroborate those of Frenk et al. and others’ in showing that the current financial investment in professional education is insufficient to meet the growing demand in global health [7, 24]. Engaging all stakeholders in global health education and training is needed in order to adequately fund professional education in the field and train the multi-disciplinary workforce required to address current global health priorities and to help foster health equity around the globe.

Increased global health training opportunities for students and faculty should be considered a priority. Short-term fellowship and training programs are available for faculty in developing economies to spend time in a developed country, such as the Fulbright Training Program, U.S. National Institutes of Health or programs funded by governments, such as Japan, These programs can provide training opportunities that do not require local university support. Such programs contribute to sharing of unique experiences and expertise from both developing and developed nations, benefitting both parties and can often lead to additional research collaborations.

Increased funding of international field placement opportunities will allow more students to gain valuable global health experience first-hand. Universities should provide competitive scholarships and placement assistance for students who want to do well-planned fieldwork abroad. While universities struggle with budget constraints that often make these programs cost-prohibitive, we feel global health practicums are a vital part of global health education and training. The value of international global health practicum opportunities as been well-documented in the literature; through such experiences students can gain crucial exposure, empathy and witness first-hand how public health works in the real world including cultural competency and language skills, increased awareness regarding health challenges in resource-constrained health systems and greater self-confidence [28, 32, 33].

In addition, financial support to cover travel costs to global health conferences may also be an important way to expose students and faculty to global health research being conducted around the globe and to meet others doing similar work. Extramural conference funding opportunities, such as conference travel grants, are also often available. Offering free global health symposiums and conferences at universities for local students and faculty might also facilitate increased interest and exposure to global health research, as well as promote networking opportunities.

## Conclusions

The discipline of global health is increasingly recognized as making an important contribution to achieving greater health equity and improving health outcomes globally. Many economies in the Pacific Rim share overlapping health challenges, concerns and goals. Instructional and institutional changes are needed to meet the growing demand for trained global health professionals from a range of disciplines. Pacific Rim universities need to develop and expand global health educational opportunities to meet the upcoming challenges in providing global health education. Training a competent global health workforce will not only help to reduce the current global burden of disease in the Pacific Rim and build capacity to tackle current and emerging health threats, but can be expected to set long-term global health priorities and influence policy on a global scale.

## Footnotes

1 Johns Hopkins and Hanoi School of Public Health are not APRU members. However, they have cooperated for many years in the APRU Global Health Program as non-member universities.
